# Pathological fracture as initial manifestation of malignancy and survival based on diagnoses: a register-based cohort study from the Swedish Fracture Register and the Swedish Cancer Register

**DOI:** 10.2340/17453674.2026.46894

**Published:** 2026-10-07

**Authors:** Jonas SUNDKVIST, Jenny GIAEVER, David WENNERGREN, Sead CRNALIC, Johan WÄNMAN

**Affiliations:** 1 Department of Diagnostics and Intervention (Orthopaedics), Umeå University, Umeå; 2 Department of Orthopaedics, Institute of Clinical Sciences, Sahlgrenska Academy, University of Gothenburg, Gothenburg, Sweden

## Abstract

**Background and purpose:**

Pathological fracture as the initial manifestation of malignancy is a clinical challenge requiring rapid treatment despite limited prognostic information. We aimed to describe definitive diagnoses and survival in patients presenting with a pathological fracture as initial manifestation of malignancy.

**Methods:**

We performed a nationwide register-based cohort study using the Swedish Fracture Register (SFR) and Swedish Cancer Register (SCR), 2014–2023. We included patients with a pathological fracture classified as unknown in the SFR at treatment. Survival was analyzed using Kaplan–Meier and Cox regression.

**Results:**

We included 319 patients with a pathological fracture. Median age was 75 years (17–100), 50% men. A definitive tumor diagnosis was established in 215 patients (67%), most commonly a hematogenous malignancy. Age was associated with mortality (HR 1.03, 95% confidence interval [CI] 1.01–1.04). Compared with hematogenous malignancies, mortality was higher for bone (HR 2.9, CI 1.2–7.2), breast (HR 2.5, CI 1.2–5.1), kidney (HR 3.3, CI 1.7–6.5), lung (HR 6.3, CI 3.7–10.5), unknown (HR 8.1, CI 4.2–15.5), other (HR 10.3, CI 5.6–19.0), and gastrointestinal malignancies (HR 10.3, CI 4.5–23.7), and for patients without SCR registration (HR 19.7, CI 11.7–33.2). Prostate cancer, sex, fracture site, and treatment were not associated with increased mortality.

**Conclusion:**

Hematogenous and lung malignancies were the most common in pathological fracture as initial manifestation of malignancy. Survival was short and varied substantially by tumor type. Age and primary tumor site were prognostic factors, emphasizing the importance of comprehensive diagnostic work-up to guide acute treatment.

A pathological fracture as the initial presentation of occult cancer is a major challenge for both orthopedic surgeons and oncologists. Treatment decisions often need to be made urgently, particularly for lower-extremity fractures where restoring ambulation needs to be achieved within a short period. However, these decisions are frequently made with limited information regarding the underlying tumor, available oncological therapies, or expected survival [1,2].

Rapid intervention is often required, as older patients who become non-ambulatory due to pain can deteriorate quickly during involuntary bed rest [3,4]. The primary goals of surgical treatment for pathological fractures are to alleviate pain and restore mobility, function, and quality of life [1,5]. The choice of surgical construct depends on the patient’s clinical status, fracture characteristics, and the risk of complications or revision surgery [1].

The extent to which osteosynthesis with screws, plates, or intramedullary nails achieves fracture union in the setting of malignancy is uncertain [6]. Such methods provide temporary stabilization that may suffice if survival is short. Conversely, when survival is expected to exceed several years, more durable procedures—such as joint replacement or megaprosthetic reconstruction—may be appropriate [7].

Survival following skeletal metastasis varies widely by tumor type [8-11]. In earlier work from our institution, we identified a subgroup of patients who presented with a pathological fracture without a known malignancy at the time of fracture [9].

We aimed to describe definitive tumor diagnoses and survival in patients presenting with a pathological fracture as the primary sign of malignancy based on 2 national patient registries, the Swedish Fracture Register (SFR) and the Swedish Cancer Register (SCR).

## Methods

### Study design

We conducted a nationwide register-based, cohort study using data from 2 national registries: the Swedish Fracture Register (SFR) and the Swedish Cancer Register (SCR). The SFR was used to identify patients who sustained a pathological fracture as the initial manifestation of malignancy, defined as having no primary tumor known at the time of fracture treatment. The SCR provided data on subsequent cancer diagnoses, including primary tumor site and classification following diagnostic work-up. Our report complies with the Strengthening the Reporting of Observational studies in Epidemiology (STROBE) guidelines.

The SFR is used by all 54 orthopedic departments in Sweden, with a reported completeness of 70–96%, depending on fracture site [12]. Reporting in the SCR is mandatory, with completeness exceeding 95% for all cancer diagnoses [13].

### Data selection

The study population included all patients with a pathological fracture registered in the SFR between 2014 and 2023. We excluded patients with a known primary tumor at the time of registration and those classified as having “other primary tumor.” The remaining cohort was cross-linked with the SCR to identify subsequent cancer diagnoses and primary tumor sites.

To ensure the pathological fracture was the primary presentation of malignancy, we excluded patients with a cancer diagnosis recorded in the SCR more than 30 days before or more than 180 days after the fracture. Entries with incomplete data in either register were also excluded. Patients with no final cancer diagnosis recorded in the SCR and a survival time of less than 180 days following the pathological fracture, in whom a complete diagnostic workup and subsequent registration in the SCR may not have been performed before death, were classified as having a pathological fracture of unknown status. Patients with a survival time exceeding 180 days who, despite adequate follow-up time, did not receive a definitive cancer diagnosis were analyzed separately due to uncertainty regarding whether the pathological fracture was caused by a malignancy.

### Patients

Patient demographics included age and sex. Fracture-related variables comprised fracture site and treatment type (surgical or non-surgical). Oncological variables included the primary tumor diagnosis, established during the subsequent diagnostic work-up.

### Formation of clusters based on ICD-10 codes

Diagnoses extracted from the SCR were grouped into clusters to facilitate statistical analysis. Clusters were based on the anatomical location of the primary tumor according to ICD‑10 codes, except for hematogenous malignancies, which were grouped separately. The final clusters were: breast, gastrointestinal, hematogenous, kidney, lung, prostate, bone malignancy, cancer of unknown primary (CUP), unknown, and other.

The codes employed were: Breast: C50.4, C50.9; Gastrointestinal: C15.5, C16.0, C17.9, C20.9, C26.0, C26.9; Hematogenous: C83.8, C84.5, C85.1, C88.0, C90.0, C90.2; Kidney: C64.0, C64.9; Lung: C34.0, C34.2, C34.3, C34.9; Prostate: C61.9; Bone malignancy: C40.0, C40.2, C41.2, C41.4; CUP: C80.9, C79.5; Unknown: Absence of an ICD‑10 in SCR; Other: C22.0, C22.1, C22.9, C25.0, C25.2, C25.9, C43.5, C44.5, C47.3, C49.2, C51.9, C53.9, C54.1, C66.9, C67.9, C73.9, C74.0, C76.3, C77.5, C77.9, C78.0, C79.8. This cluster included tumors of the liver and gallbladder, pancreas, melanoma, skin and soft tissue, peripheral nervous system, urogenital tract, thyroid, adrenal glands, abdomen, and unspecified metastases.

### Statistics

Statistical analyses were performed using IBM SPSS Statistics version 30.0 (IBM Corp, Armonk, NY, USA). Continuous variables are presented as medians (range) and categorical variables as frequencies and percentages. Survival was estimated using the Kaplan–Meier method, and median survival times were reported with 95% confidence intervals (CI). Cox proportional hazards regression models were used to calculate hazard ratios (HR) for mortality, adjusted for age, sex, primary tumor site, and fracture site. The proportional hazards assumption was verified using log–log plots. Logistic regression was used to examine the association between fracture treatment (surgical vs non-surgical) and fracture site. The primary outcome was overall survival (OS), defined as the time from fracture to death or end of follow-up (censoring). Two-sided P values < 0.05 were considered statistically significant.

### Ethics, data sharing plan, use of AI, funding, and disclosures

This study was approved by the Swedish Ethical Review Authority (No. 2023-07809-01). All data were pseudonymized before analysis to ensure patient confidentiality.

The dataset analyzed in this study is not publicly available, as the study was approved on the condition that the confidentiality of patient data be ensured. While the authors are willing to share data, legal restrictions prevent public data sharing in accordance with the Swedish Public Access to Information and Secrecy Act (Offentlighets- och sekretesslag [2009:400]), Chapter 21, Section 7, and Chapter 25, Section 1 (https://www.riksdagen.se/sv/dokument-lagar/dokument/svensk-forfattningssamling/offentlighets--och-sekretesslag-2009400_sfs-2009-400).

Generative AI was employed solely for linguistic refinement, grammatical editing, and figure editing. The authors maintain full responsibility for the original writing of the manuscript and the integrity of the data analysis, neither of which involved AI assistance.

No external funding was received. JS is a board member of the SFR. The authors declare no other competing interests. Complete disclosure of interest forms according to ICMJE are available on the article page, doi: 10.2340/17453674.2026.46894

## Results

### Patients

2,480 pathological fractures were identified in the SFR. After excluding cases with a known primary tumor (n = 1,867), 613 remained. After removal of duplicates 563 remained. 6 patients were excluded due to incomplete registration in the SFR, leaving 557 patients for cross-linking with the SCR. 295 patients had a final cancer diagnosis in the SCR while 262 had no match in the SCR. Among these, 86 had short postoperative survival (< 180 days) and were included as unknown, while 176 patients had a survival > 180 days and were analyzed separately due to uncertainty of malignancy as a cause of the pathological fracture. A further 62 patients were excluded because their cancer diagnosis was recorded in the SCR more than 30 days prior to the fracture or > 180 days after the fracture. The final analysis included 319 patients with 319 pathological fractures as the index event (Figure 1).

**Figure 1:**
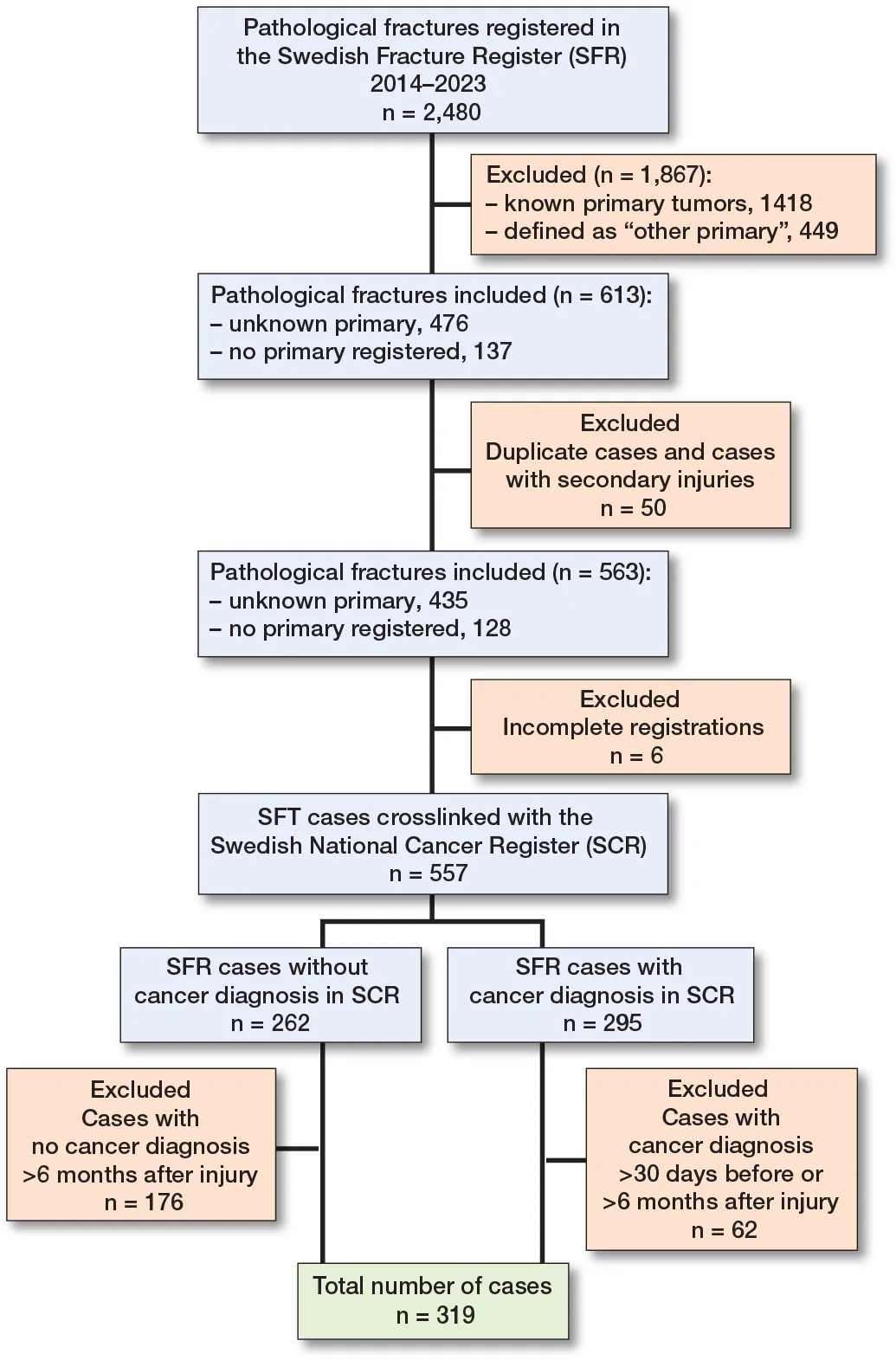
Flowchart illustrating the data selection process.

There was an even sex distribution, with 159 women (50%) and 160 men (50%). Median age was 75 years (17–100) (Table 1).

**Table 1:** Clinical characteristics of 319 patients with pathological fractures, stratified by primary tumor site

Site	Age median (range)	Sex		Lower extremity	Pelvis/acetabulum	Spine	Upper extremity	Total
		Male	Female					
Breast	70 (46–90)	–	14	9 ^**d**^	–	–	5	14
Gastrointestinal	69 (38–81)	6	4	3	1 ^**e**^	4 ^**f**^	2	10
Hematogenous	74 (33–96)	37	29	19 ^**g**^	1	14 ^**h**^	32 ^**i**^	66
Kidney	71 (56–93)	12	6	6	2	1	9	18
Lung	73 (30–95)	18	34	25	1	7 ^**j**^	19 ^**k**^	52
No SCR ^**a**^	78.5 (50–100)	42	44	45	4	8	29 ^**l**^	86
Other ^**b**^	74 (22–88)	12	15	18 ^**m**^	–	3	6 ^**n**^	27
Prostate	81.5 (56–92)	16	–	12	1	1	2	16
Bone	60.5 (17–86)	7	5	9	–	1	2	12
Unknown ^**c**^	77 (52–87)	10	8	12	–	1	5	18
Total	75 (17–100)	160	159	158	10	40	111	319

a Includes patients with fractures registered in the Swedish Fracture Register (SFR) without registration in the Swedish Cancer Register (SCR) < 6 months after injury.

b Includes primaries in abdomen, adrenal glands, liver and gallbladder, melanoma, metastasis with no further specification, pancreas, peripheral nerve sheath, skin and soft tissue, thyroid, and urogenital tract.

c Includes confirmed cancer of unknown primary and skeletal metastasis with no reported primary.

d 1 of these patients had multiple femoral fractures, 2 in total.

e 1 of these patients also had spinal fractures in 2 different segments.

f 1 of these patients had multiple spinal fractures in 3 different segments.

g 2 of these patients also had a hip fracture.

h 1 of these patients had multiple spinal fractures in 2 different segments.

i 2 of these patients had multiple humoral fractures, 2 in total.

j 1 of these patients had multiple spinal fractures in 2 different segments.

k 1 of these patients also had a clavicular fracture.

l 1 of these patients also had a scapular fracture. Another also had a femoral fracture.

m 1 of these patients also had a spinal fracture.

n 1 of these patients had multiple humoral fractures, 2 in total.

### Fracture characteristics

The most frequent fracture site was the lower extremities (50%), followed by the upper extremities (35%), the spine (13%), and the pelvis/acetabulum (3%) (see Table 1). Non-surgical treatment was administered to 91 patients (29%), while 204 (64%) underwent surgical intervention; treatment data was missing for 24 patients (7.5%) (Table 2 and Supplementary Table S1). Among those initially treated non-surgically, 8 (8%) later required surgery. 6 patients (3%) underwent revision surgery, and 1 required a second revision.

**Table 2:** Choice of treatment in relation to primary tumor site

Treatment	Breast	Gastrointestinal	Hematogenous	Kidney	Lung	No SCR ^a^	Other ^b^	Prostate	Bone	Unknown ^c^	Total
Non-surgical treatment	2	5	20	4	18	27	7	3	3	2	91
Surgical first treatment	12	4	39	12	31	54	17	13	6	16	204
Excision arthroplasty	1	–	–	–	–	2	–	–	–	–	3
External fixation	–	–	–	–	1	–	–	–	–	–	1
Osteosynthesis	9	2	27	6	23	35	14	4	4	11	135
Prosthesis	2	2	7	5	5	15	3	8	1	4	52
Spinal surgery											
with decompression	–	–	–	–	2	1	–	–	–	1	4
with fusion	–	–	–	1	–	–	–	–	–	–	1
with both	–	–	5	–	–	1	–	1	1	–	8
No treatment registered	–	1	7	2	3	5	3	–	3	–	24
Total	14	10	66	18	52	86	27	16	12	104	319

a–c See Table 1.

11 patients (3%) died within the first week after presentation. Of these, 7 received no surgical treatment, 3 underwent primary surgery, and 1 had no treatment recorded. None of these 11 patients underwent secondary surgery. Their median age was 86 years (61–100 years).

### Oncological findings

Of the 319 patients, 233 (73%) received a definitive primary tumor diagnosis in the SCR. The remaining 86 patients (33%) were recorded as having an unknown primary tumor.

Among patients with an identified primary tumor, the most common diagnoses were hematogenous malignancies (21%), followed by lung (16%), kidney (6%), prostate (5%), breast (4%), and gastrointestinal tumors (3%). Bone malignancies accounted for 4%, while other miscellaneous tumors comprised 9% (Table 3).

**Table 3:** Survival at 30 days, 90 days, and 1 year, stratified by primary tumor site

Site	Total number	Alive at 30 days	Alive at 90 days	Alive at 1 year
Breast	14	14	13	10
Gastrointestinal	10	10	3	2
Hematogenous	66	65	61	53
Kidney	18	16	15	8
Lung	52	51	33	10
No SCR ^**a**^	86	51	24	0
Other ^**b**^	27	23	12	4
Prostate	16	16	15	12
Bone	12	12	11	9
Unknown ^**c**^	18	13	6	2
Total, n (%)	319	271 (85)	195 (61)	110 (35)

a–c See Table 1.

### Survival

Overall median survival was 141 days (CI 115–168). The 30-day survival rate was 85%, the 90-day survival rate was 61%, and the 1-year survival rate was 35% (see Table 3). Kaplan–Meier analysis demonstrated significant differences in survival rates across primary tumor sites (Figure 2).

**Figure 2:**
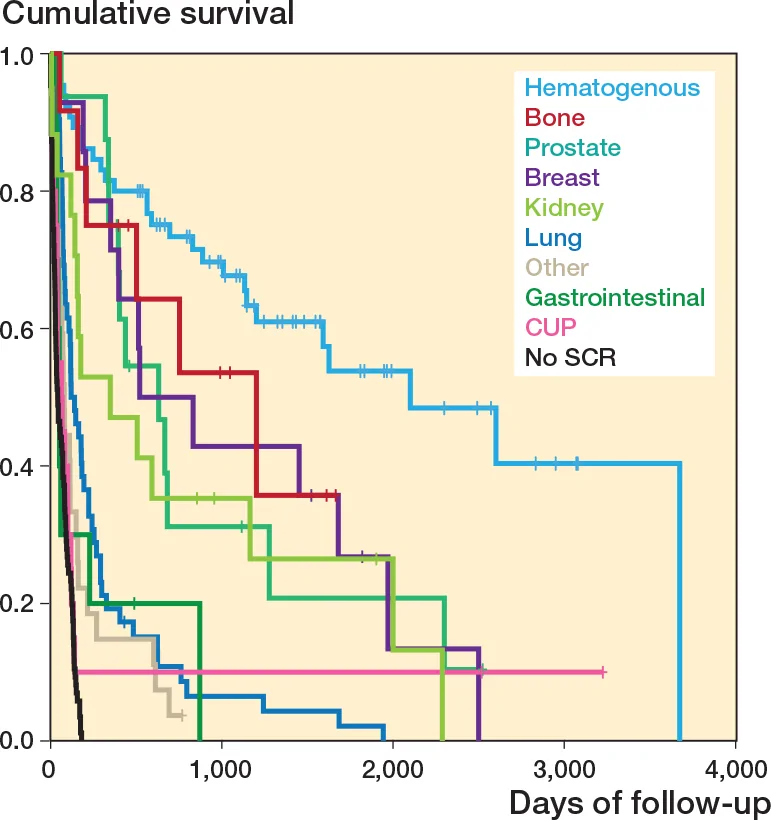
Kaplan–Meier survival curves illustrating overall survival (days) following the diagnosis of a pathological fracture, stratified by primary tumor site.

The longest median survival was observed in patients with hematogenous malignancies (2,102 days, CI 1,116–3,087), followed by bone malignancies (1,203 days, CI 343–2,063), prostate cancer (632 days, CI 329–935), breast cancer (522 days, CI 0–1,105), kidney cancer (178 days, CI 0–567), lung cancer (121 days, CI 7–170), other tumors (81 days, CI 64–98), and gastrointestinal tumors (52 days, CI 40–64). Patients with CUP (ICD 80.9, 79.5) had a median survival of 41 (CI 0–91) and patients categorized as unknown (with no ICD in the SCR) had a median survival of 42 (CI 20–64).

Median survival did not differ significantly by fracture site (P = 0.1): 224 days (CI 0–616) for the spine, 141 days (CI 73–109) for lower-extremity, 138 days (CI 93–183) for upper-extremity, and 40 days (CI 0–93) for pelvis/acetabulum fractures.

In the subgroup analysis of patients with survival > 180 days and no recorded cancer diagnosis in the SFR, median survival was 1,732 days (CI 1,254–2,209) (Supplementary Figure S1).

### Survival predictors

In the unadjusted Cox regression model, older age, all primary tumor sites except bone malignancy, and lower-extremity fractures were significantly associated with mortality. In the adjusted analysis, increasing age remained associated with mortality (HR 1.03, CI 1.01–1.04). Using hematogenous malignancies as reference, mortality was higher for bone (HR 2.9, CI 1.2–7.2), breast (HR 2.5, CI 1.2–5.1), kidney (HR 3.3, CI 1.7–6.5), lung (HR 6.3, CI 3.7–10.5), other (HR 10.3, CI 5.6–19.0), gastrointestinal (HR 10.3, CI 4.5–23.7), and unknown malignancies (HR 8.1, CI 4.2–15.5), and among patients without SCR registration (HR 19.7, CI 11.7–33.2). Prostate cancer, sex, fracture site, and fracture treatment were not independently associated with mortality (Table 4).

**Table 4:** Cox proportional hazards regression analysis of factors associated with overall survival. Hazard ratios (HR) > 1 indicate an increased risk of mortality relative to the reference category. Univariate analysis demonstrates a significant association with increasing age and across all primary tumor sites, except for bone malignancy. In the multivariate (adjusted) model, significance is maintained for increasing age and all primary tumor sites, except for prostate cancer

Variable	Crude HR (CI)	P value	Adjusted ^d^ HR (CI)	P value
Age	1.0 (1.0–1.0)	< 0.01	1.0 (1.0–1.0)	< 0.01
Sex				
Male	Reference		Reference	
Female	1.2 (0.9–1.5)	0.2	1.0 (0.8–1.3)	0.9
Primary tumor		< 0.01		< 0.01
Hematogenous	Reference		Reference	
Bone	1.6 (0.6–3.8)	0.3	2.9 (1.2–7.2)	0.02
Prostate	2.2 (1.1–4.3)	0.02	1.8 (0.9–3.8)	0.1
Breast	2.2 (1.1–4.4)	0.02	2.5 (1.2–5.1)	0.01
Kidney	3.2 (1.7–5.9)	< 0.01	3.3 (1.7–6.5)	< 0.01
Lung	6.2 (3.9–10.0)	< 0.01	6.3 (3.7–10.5)	< 0.01
Gastrointestinal	8.2 (3.8–17.5)	< 0.01	10.3 (4.5–23.7)	< 0.01
No SCR ^**a**^	19.5 (12.0–31.7)	< 0.01	19.7 (11.7–33.2)	< 0.01
Other ^**b**^	8.8 (5.1–15.3)	< 0.01	10.3 (5.6–19.0)	< 0.01
Unknown ^**c**^	6.7 (3.6–12.5)	< 0.01	8.0 (4.2–15.5)	< 0.01
Segment		0.1		0.7
Spine	Reference		Reference	
Upper extremity	1.2 (0.8–1.9)	0.4	0.8 (0.5–1.4)	0.5
Lower extremity	1.5 (1.0–2.3)	0.04	1.0 (0.6–1.6)	0.9
Pelvis/acetabulum	1.8 (0.8–3.8)	0.1	1.2 (0.5–2.7)	0.7
Treatment				
Non-surgical	Reference		Reference	
Primary surgery	1.0 (0.8–1.3)	> 0.9	0.8 (0.6–1.1)	0.2

CI = 95% confidence interval.

a–c See Table 1.

d Adjusted for age, sex, primary tumor, segment, and treatment.

### Factors associated with treatment choice

Logistic regression demonstrated that fracture site was the only variable significantly associated with treatment choice in both crude and adjusted models. Age, sex, and primary tumor showed no significant association with the decision for surgical vs non-surgical intervention (Supplementary Table S2).

## Discussion

We aimed to describe definitive diagnoses and survival in patients presenting with a pathological fracture as the initial manifestation of malignancy in a nationwide, register-based study. The primary finding was that hematogenous and lung malignancies were the most common causes of pathological fractures. A pathological fracture as a sentinel event was associated with markedly heterogeneous survival, ranging from only a few weeks to several years depending on the primary tumor site.

Although overall survival was poor, with a median of 4.5 months, certain subgroups achieved prolonged survival, sometimes extending over several years. Notably, fracture site was the only factor significantly associated with treatment choice, whereas age, sex, and primary tumor site were not.

### Fracture characteristics

Our finding that the lower extremities were the most frequent site of pathological fractures diverges from the established literature [14-16]. One explanation may relate to the distinction between reporting metastatic sites and reporting pathological fractures, as the proportion of bone metastases that progress to fracture remains uncertain [17]. Another likely explanation stems from registry coverage. The SFR does not collect data on rib fractures; the completeness of pelvic fracture registration is below 40%; and the completeness of spinal fracture registration is unknown [12]. In addition, most surgically treated spinal tumors in Sweden are reported to the Swedish Spine Register (Swespine), potentially contributing to the underrepresentation of spinal fractures in the SFR.

### Survival analysis

Overall median survival was 141 days, with pronounced differences observed across primary tumor groups. Patients with hematogenous malignancies had the longest survival, with a median of approximately 5.8 years, whereas those classified as having an unknown primary tumor or CUP had the shortest survival at only 42 days. The long survival observed in hematogenous malignancies supports findings that survival is influenced by the potential for further oncological treatment following initial fracture management [10,18].

Studies have reported 30-day survival rates of approximately 90% and 1-year survival rates ranging from 45–50% in patients with skeletal metastases [9,10,18,19]. In our cohort, the 30-day survival rate was 85%, while the 1-year survival rate was notably lower at 35%. This 1-year survival varied widely across groups, ranging from 80% in hematogenous malignancies to a mere 2% in unknown primaries.

Survival for prostate (632 days), breast (522 days), and lung cancer (121 days) was longer than reported in 1 comparable study (20), suggesting that identifying hormone-therapy-naive patients offers opportunities for effective oncological treatment. In selected patients, such therapy may meaningfully improve survival.

In our analysis, age and primary tumor site were significantly associated with survival, whereas fracture site, sex, and fracture treatment were not. This observation contrasts with a study reporting a significant association between fracture treatment and survival [21].

Our findings underscore the importance of identifying patients with a more favorable prognosis, as certain malignancies remain amenable to effective oncological therapy and prolonged survival—as illustrated by the hematogenous group in this cohort. In contrast, patients diagnosed with CUP or skeletal metastasis without an identified primary tumor had a median survival of only 42 days, substantially shorter than that reported in studies of unknown primary tumors [22]. This pronounced mortality difference may reflect that a pathological fracture represents a later stage of disease progression compared with metastatic lesions in which skeletal integrity is preserved. Additionally, some patients in this subgroup were likely in poor clinical condition, making an extensive diagnostic work-up ethically inappropriate; in such cases, prioritizing symptom relief and palliative care was more aligned with patient needs. A further subgroup of patients with long survival (> 180 days) without a recorded cancer diagnosis likely represents a clinically heterogeneous population, including both non-malignant fractures and cases with occult or unregistered malignancy. The prolonged survival in this group suggests that not all pathological fractures in this setting are attributable to cancer, highlighting the diagnostic uncertainty inherent in register-based classification. From a surgical perspective, this underscores the importance of structured diagnostic evaluation prior to definitive fracture management.

### Factors associated with the choice of fracture treatment

In our cohort, the anatomical site of the pathological fracture was the only factor associated with the choice of surgical treatment. Nevertheless, given the considerable heterogeneity in survival across malignancy groups, a rapid diagnostic evaluation remains essential to support more individualized treatment decisions. Establishing the primary tumor type allows clinicians to better balance expected survival and tumor biology against the anticipated benefits of surgical intervention—ultimately enabling more patient-specific and ethically aligned fracture management.

### Strengths

The major strength of this study is the large sample size provided by the nationwide coverage of the SFR. In addition, the ability to combine data from both the SFR and SCR—facilitated by the unique personal identification number assigned to all Swedish residents—allowed for robust linkage of fracture, oncological, and survival data.

### Limitations

Miscoding, missing registrations, and reporting variability remain inherent challenges. Additionally, the dataset lacks granular information regarding the diagnostic work-up. We identified 3 groups with an unknown or unconfirmed primary malignancy: CUP (C80.9), skeletal metastasis without a specified primary tumor (C79.5), and no SCR entry with death within 180 days of fracture. The last group may have been too ill for diagnostic work-up. Patients without a confirmed cancer diagnosis who survived longer than 180 days were analyzed separately. Misclassification remains possible. In addition, potential underreporting and delays in registry coding may have affected case classification. Sweden maintains mandatory national guidelines for the evaluation of unknown primary tumors, and established methods identify primary malignancies in the majority of cases [10,18,23]. However, the level of adherence to these protocols could not be assessed. Although the SCR is known for its high coverage and completeness, patients with a pathological fracture as initial manifestation of malignancy may be susceptible to underreporting and misclassification due to short survival or limited diagnostic work-up.

Furthermore, the data sources did not include information on patients’ comorbidities; however, existing evidence suggests that comorbidities do not significantly influence survival following fracture treatment [20]. Finally, neither the SFR nor the SCR contains information on adjuvant oncological therapies, which may meaningfully affect survival outcomes and therefore represent an additional constraint in interpreting the results.

### Conclusion

Overall survival following a pathological fracture as the first indication of systemic disease was short, although appreciable variance was observed across tumor types. Age and the anatomical distribution of the primary tumor were associated with survival outcomes.

These findings suggest that patients presenting with a pathological fracture as the first sign of malignancy may benefit from a rapid, targeted diagnostic work-up to guide appropriate fracture management and align treatment with the expected prognosis, notably identification of the hematogenous malignancies with a longer survival.

### Supplementary data

Tables S1, S2, and Figure S1 are avaiable as supplementary data on the article home page, doi: 10.2340/17453674.2026.46894

## Supplementary Material



## Notes

JS and JW conceived and designed the study. JW and JG performed the statistical analyses. JS and JW supervised JG. JS and JG drafted the manuscript. JS, JG, DW, SC, and JW contributed to data interpretation, revised the manuscript, and approved the final version.

The authors thank the staff of the Swedish Fracture Register and the Swedish Cancer Register for their support. They are also grateful to the physicians and administrative staff at orthopedic departments across Sweden for their invaluable contributions to the maintenance and accuracy of these national registers. Further, they wish to thank Leslie Shaps for the professional language review.

Handling co-editors: Bart Pijls and Robin Christensen

*Acta* thanks Christian Kveller and Martyn Parker for help with peer review of this manuscript.
