# Supplementary material for: Pathological fracture as initial manifestation of malignancy and survival based on diagnoses: a register-based cohort study from the Swedish Fracture Register and the Swedish Cancer Register
Source: Acta Orthop. 2026 Oct 7;97:727–33. doi: 10.2340/17453674.2026.46894 (PMC13644678; doi:10.2340/17453674.2026.46894)
Supplement: Supplementary file 1 [file ActaO-97-46894-s1.pdf]

Supplementary data

Supplementary Table S1. Choice of treatment in relation to fracture site

Type	Acetabulum	Clavicle	Femur	Forearm	Hip	Humerus	Pelvis	Scapula	Spine	Tibia	Total
Non-surgical treatment	3	14	6	2	2	34	6	1	18	5	91
Surgical first treatment	–	–	42	1	91	52	–	–	13	5	204
Excision arthroplasty	–	–	–	–	3	–	–	–	–	–	3
External fixation	–	–	1	–	–	–	–	–	–	–	1
Osteosynthesis	–	–	40	1	39	50	–	–	–	5	135
Prosthesis	–	–	1	–	49	2	–	–	–	–	52
Spinal surgery											
with decompression	–	–	–	–	–	–	–	–	4	–	4
with fusion	–	–	–	–	–	–	–	–	1	–	1
with both	–	–	–	–	–	–	–	–	8	–	8
No treatment registered	1	–	1	1	5	6	–	-	9	1	24
Total	4	14	49	4	98	92	6	1	40	11	319

Supplementary Table S2. Binary logistic regression analysis of factors associated with the choice of surgical vs non-surgical treatment. Odds ratios (ORs) > 1 indicate an increased probability of receiving surgical intervention. In both univariate and multivariate (adjusted) models, the anatomical fracture site was the only covariate significantly associated with the treatment choice

Variable	Crude		Adjusted [a]	
	OR (CI)	P value	OR (CI)	P value
Age	1.0 (1.0–1.0)	0.7	1.0 (1.0–1.0)	0.3
Sex				
Male	Reference		Reference	
Female	1.4 (0.9–2.3)	0.2	1.5 (0.8–2.8)	0.2
Primary tumor		0.4		0.4
Hematogenous	Reference		Reference	
Bone	1.0 (0.2–4.5)	0.9	0.3 (0.1–1.7)	0.2
Prostate	2.2 (0.6–8.7)	0.3	1.6 (0.2–9. 9)	0.6
Breast	3.1 (0.6–15.1)	0.2	1.3 (0.2–7.5)	0.8
Kidney	1.5 (0.4–5.4)	0.5	2.0 (0.5–9.0)	0.4
Lung	0.9 (0.4–2.0)	0.8	0.5 (0.2–1.3)	0.1
Other [b]	1.3 (0.4–3.5)	0.	0.5 (0.1–1. 6)	0.2
Gastrointestinal	0.4 (0.1–1.7)	0.2	0.3 (0.1–2.1)	0.3
Unknown [c]	4.1 (0.9–19.6)	0.08	2.4 (0.4–13.5)	0.3
No SCR [d]	1.0 (0.5–2.1)	0.9	0.7 (0.3–1.6)	0.4
Segment		< 0.01		< 0.01
Spine	Reference		Reference	
Upper extremity	1.4 (0.6–3.2)	0.4	1.2 (0.5–2.9)	0.6
Lower extremity	14.7 (5.9–36.6)	< 0.01	14.8 (5.6–38.9)	< 0.01
Pelvis/acetabulum	0.0 (0.0–0.0)		0.0 (0.0–…)	

CI = 95% confidence interval.

[a] Adjusted for age, sex, primary tumor, and segment.

[b] Includes primaries in abdomen, adrenal glands, liver and gallbladder, melanoma, metastasis with no further specification, pancreas, PNS, skin and soft tissue, thyroid, and urogenital tract.

[c] Includes confirmed CUP and skeletal metastasis with no reported primary.

[d] Includes patients with fractures registered in the Swedish Fracture Register (SFR) without registration in the Swedish Cancer Register (SCR) < 6 months after injury.

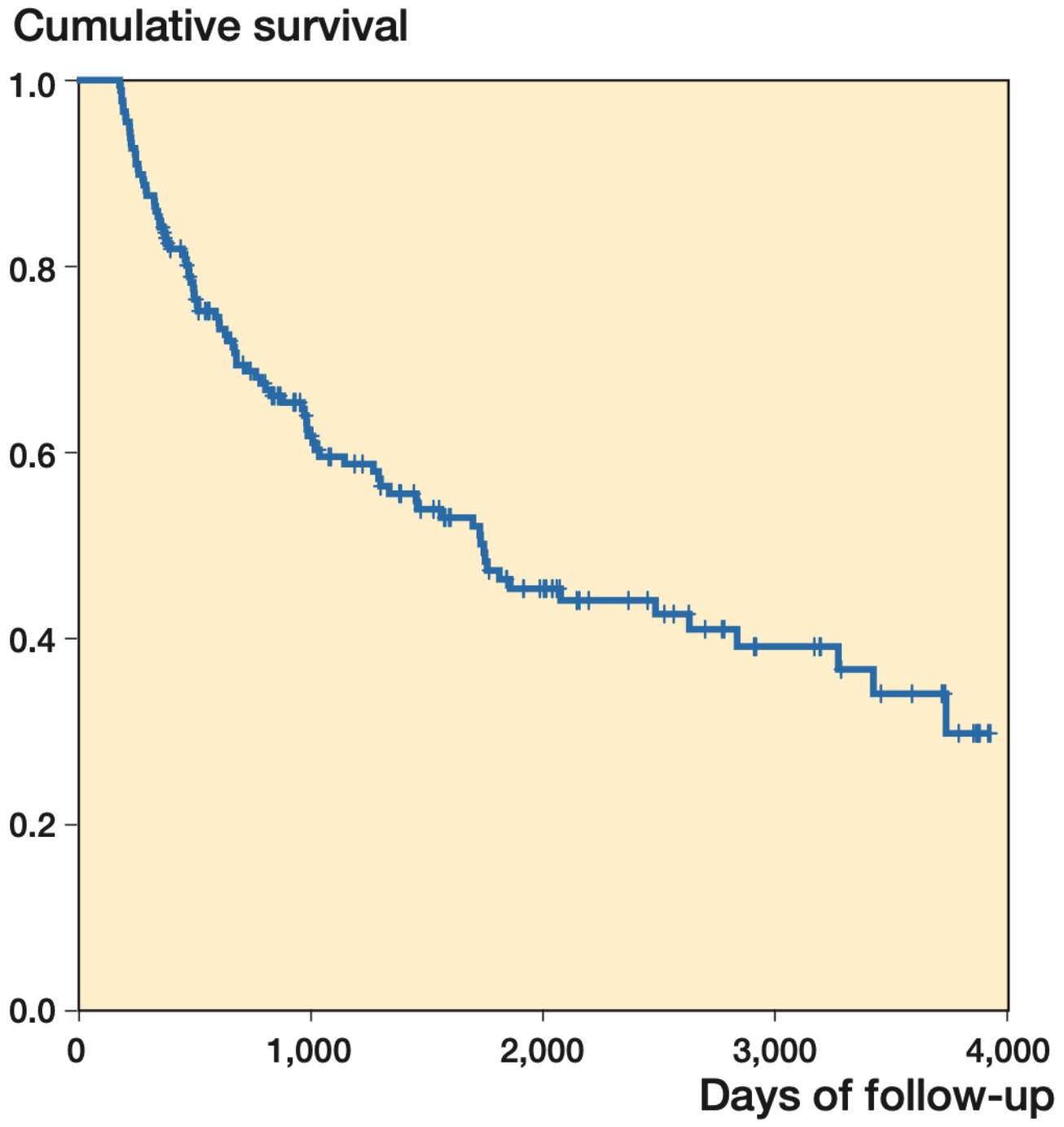

Supplementary Figure S1. Kaplan–Meier curve of survival (days) after pathological fracture in 176 excluded patients without a registered cancer diagnosis in the Swedish Cancer Register and survival more than 6 months after injury.
